# Seroprevalence and genetic characterization of *Toxoplasma gondii* in cancer patients in Anhui Province, Eastern China

**DOI:** 10.1186/s13071-015-0778-5

**Published:** 2015-03-15

**Authors:** Lin Wang, Liu-yuan He, De-di Meng, Zhao-wu Chen, He Wen, Gong-si Fang, Qing-li Luo, Kai-quan Huang, Ji-long Shen

**Affiliations:** Clinical Laboratory, the First Affiliated Hospital of Anhui University of Chinese Medicine, 230031 Hefei, Anhui Province China; Department of Parasitology, Provincial Laboratory of Microbiology & Parasitology and the Key Laboratory of Zoonoses Anhui, Anhui Medical University, 230032 Hefei, Anhui Province China; Department of Medical Technology, Anhui Medical College, 230601 Hefei, Anhui Province China; Clinical Laboratory, Anhui Tumour Hospital, West Campus of Affiliated Provincial Hospital of Anhui Medical University, 230022 Hefei, Anhui Province China; Clinical Laboratory, the First People’s Hospital, 230061 Hefei, Anhui Province China

**Keywords:** *Toxoplasma gondii*, Cancer patients, Seroprevalence, Genotyping, China

## Abstract

**Background:**

Recent studies have indicated the predominance of *Toxoplasma gondii* genotype Chinese 1 in animals in China. However, little is known of the genetic features of the parasite in humans. This study aims to determine the prevalence of anti-*T. gondii* antibodies based on which the genetic character of the parasite was identified in cancer patients in China.

**Methods:**

A total of 1014 serum samples with malignant neoplasms were collected from six tertiary-care hospitals (HAUCM, APH, HAMU, XAH, FHH and HBMC) from January, 2012 to August, 2013. Antibodies against *T. gondii* were examined by enzyme-linked immunosorbent assay (ELISA). Blood samples were subsequently used for PCR assay to detect *T. gondii* DNA (*gra6*). The DNA positive samples were subjected to genotyping using a multiplex multilocus nested PCR-RFLP at 10 loci, including *sag1*, *sag2, sag3, btub, gra6, l358, c22-8, c29-2, pk1* and *apico*. Samples from the patients were anonymous and only data with regard to age and gender was available at sample collection.

**Results:**

Overall, 8.38% (85/1014) of the examined patients showed positive antibodies against *T. gondii*. Among them, 61 (6.02%) were seropositive only for IgG, 16 (1.58%) were only for IgM, and 8 (0.79%) were found to be positive for both IgG and IgM. The seroprevalence of antibodies to *Toxoplasma* ranged from 5.8% to 11.0%, without regional difference (*χ*^2^ = 4.764, *P* = 0.445). No significant differences of the positive rates of *T. gondii* infection were noted in genders (male, 8.96%; female, 7.45%) (*χ*^2^ = 0.707, *P* = 0.400) and in ages (*χ*^2^ = 1.172, *P* = 0.947). Of 1014 DNA samples, 36 (3.55%) were positive for *T. gondii* by nested PCR at *gra6* locus and nine gave rise to complete genotyping results. All samples with achieved PCR-RFLP genotyping showed a common genetic character of type Chinese 1 (ToxoDB#9).

**Conclusion:**

Seroprevalence of toxoplasmosis in immunosuppressed individuals is rarely reported in China and we presented a positive rate of 8.38% in cancer patients. *Toxoplasma* genomic DNA genotyping demonstrated a common genetic character of Chinese 1, indicating a possible pathogenic origin of animals in human infection.

## Background

*Toxoplasma gondii* is a worldwide protozoan parasite that can infect virtually all warm-blooded animals, including humans. It is prevalent in most areas of the world and up to one-third of the human population is chronically infected, with an endemicity from around 10% to 70% and the prevalence is high in warmer and humid areas [1,2]. Food-borne transmission of *T. gondii* is considered to be the most important route for human infection [3,4], which occurs through the ingestion of raw or inadequately cooked meat containing tissue cysts, or of food or water contaminated by oocysts shed by felids. Moreover, infection acquired during pregnancy can be transmitted to the foetus, sometimes leading to serious consequences [5]. *T. gondii* infection is currently incurable because the parasite can change from the rapidly replicating tachyzoite stage to the dormant bradyzoite stage, and the latter is impervious to host immunity and also to drugs. Toxoplasmosis is fatal in the immunocompromised individuals such as cancer patients with chemotherapy, HIV/AIDS patients and organ transplant recipients [6-8]. Although the infection is usually believed to be harmless and have a benign course in immunocompetent persons, it could be indirectly responsible for deaths due to its effects on the traffic and workplace accidents, and also suicides. Moreover, latent toxoplasmosis is probably one of the most important risk factors for schizophrenia [9]. China is the first-most populous nation in the world (National Bureau of Statistics 2010) and a high rate of neoplasmas. Clinical toxoplasmosis in malignant patients in China is of great public health concern, since it can lead to the active parasitemia and life-threatening disease due to the rupture of pre-existent cysts, contributing to worsening of the clinical condition [10]. Therefore, it becomes essential to investigate the prevalence and genetic structure of *T. gondii* in cancer patients of China.

The frequency of seropositivity of *T. gondii* varies in different countries or even in different areas of a given country. Prevalence below 30% was observed in USA, Northern Europe, and South East Asia [11,12], while above 60% in the regions of tropical African and Latin America [13,14]. Moreover, *T. gondii* infection ranges from 8.8% to 37.3% in women of fertile age in the Indian subcontinent [15], and from 5.1% to 16.4% in populations of Kyrgyzstan, respectively [16]. Walle *et al.* [7] from Ethiopia reported the positive rates of 87.4% and 10.7% of anti-*Toxoplasma* IgG and IgM, respectively. Previous investigation in Brazil indicated that toxoplasmic encephalitis might reach up to 40% in patients with AIDS, among them 10-30% died [17]. However, little is known in the scientific community about the epidemiology of *T. gondii* and the parasite genotypes in human populations and, particularly, in cancer patients of China.

The genetic diversity of *T. gondii* varies in different geographical regions and hosts. In North America and Europe, *T. gondii* is highly clonal and consists of three distinct lineages (types I, II and III). Type I strains are highly virulent to mice. The type II and III lineages are widespread throughout all continents and dominate in North America, Europe and Africa, meanwhile, type II strains are the most prevalent cause of human toxoplasmosis in both congenital infection and AIDS patients in North America and Europe [18-20]. In contrast, genetic characterization of isolates from human patients and animals in South America are genetically and biologically diverse [21], and severe toxoplasmosis in immunocompetent human patients is often associated with atypical genotypes [22]. We have previously identified limited genotypes of isolates from stray cats, pigs, sheep and chickens in China, and genotype ToxoDB#9 (termed as Chinese 1) is widespread and is likely the major *T. gondii* lineage circulating in animals and humans in China [23,24]. So far, neither detailed information on seroepidemiology of *Toxoplasma* in immunocompromised patients nor precise approaches concerning the genetic features of *T. gondii* isolates from humans have been uncovered in China. Genotyping studies of *T. gondii* in cancer patients may help reevaluate the population genetic structure, population biology and pathogenesis of this important zoonotic pathogen in China.

Herewith we examined the seroprevalence of anti-*T. gondii* antibodies, as well as the population structure of this parasite in cancer patients, and hope to provide baseline data for the implementation of effective strategies for the control and prevention of *T. gondii* infection in China.

## Methods

### Ethical aspects

This study was approved by the Institutional Review Board (IRB) of the Institute of Biomedicine at Anhui Medical University (Approval No: 2012012). Participation in the study was voluntary without incentives. Both studies were carried out in accordance with good clinical practices; the purpose and procedures of the study were explained to all participants, and a written informed consent was obtained from each participating patient.

### Participants and serum sample preparation

A total of 1014 inpatients and outpatients were enrolled in the study from 6 representative tertiary-care hospitals (The First Hospital of Anhui University of Chinese Medicine (HAUCM), Anhui Provincial Hospital (APH), the First Hospital of Anhui Medical University (HAMU), Xinan Hospital (XAH), the First Hospital of Hefei (FHH) and the First Hospital of Bengbu Medical College (HBMC)) in Anhui province, Eastern China, from May 2012 to August 2013. One sample from each patient was collected and the information such as gender and age were also obtained and matched. Approximately 5 ml of venous blood samples were drawn from each patient with informed consent. All the blood samples were labeled individually and cooled with ice packs to maintain the temperature at 4°C during transport to the laboratory. Blood samples were centrifuged and sera were recovered and transferred to 1.5 ml centrifuge tubes. The serum samples were stored at −80°C until tested for *T. gondii* antibodies.

### Immunological test for *T. gondii* antibodies

All serum samples were analyzed by qualitative and quantitative methods for anti-*Toxoplasma* IgG and IgM antibodies by enzyme-linked immunosorbent assay (ELISA) kits commercially available (Haitai Biological Pharmaceuticals Co., Ltd, Zhuhai, China), following the manufacturer’s instructions. Positive, negative, critical and blank controls supplied with the kit were included in each testing plate. In brief, sera diluted 1:100 were incubated in a *T. gondii* antigen-coated 96-well plate at 37°C for 30 min, and the plate was washed five times, then a drop of diluted HRP-labelled conjugate was added to each well. After a final washing, “A” and “B” solutions were added that are available in the kit and incubated at 37°C for 15 min. The optical density (OD) values of the test sera were corrected according to blank controls, and OD values were read using an automated microplate reader (Bio-Tek, Vermont, USA). The threshold value was determined by the mean of 3 critical controls in each test. A result equal to or greater than threshold values was considered positive.

### DNA extraction and *Toxoplasma gra6* amplification

Genomic DNA was extracted from the whole blood samples using the QIAamp Mini DNA kit (Qiagen, Hilden, Germany) according to the manufacturer’s instructions. The DNA samples were tested by nested PCR of the GRA6 gene. Briefly, 1.5 μl of DNA template was add to a final volume of 25 μl PCR mixture consisting of 12.5 μl PCR *Premix Taq* (TaKaRa, Dalian, China), and 1.5 μl of each of the outer primers. The amplification steps included a first cycle of denaturation at 94°C for 5 min, 35 cycles of denaturation at 94°C for 30 s, annealing at 55°C for 60 s, and extension at 72°C for 90 s, and a final extension step at 72°C for 10 min. The resulting products were diluted with equal an volume of nuclease-free water and then used as template for the nested PCR with inner primers in a total volume of 25 μl under the similar program. Each PCR set included a positive control of DNA extracted from *T. gondii* RH strain and a negative control of nuclease-free water. The PCR-generated products and molecular weight markers were subjected to electrophoresis on a 1.5% agarose gel, stained with ethidium bromide, and visualized under UV transilluminator (Hema, Zhuhai, China).

### Genetic characterization of *T. gondii* in positive DNA samples

DNA samples that were positive for *gra6* were subsequently genotyped by multi-locus PCR-RFLP using the genetic loci *sag1, sag2, sag3, btub, gra6, c22-8, c29-2, l358, pk1 and apico* [25]. Briefly, pre-amplification was carried out using a set of mixed outer primers in a single reaction. Then, multiplex PCR-amplified products were diluted 1:1 with deionized water, and then used for nested PCR amplifications with inner primers for each locus, respectively. Eight genotype references of *T. gondii* strains were set up as positive controls including GT1, PTG, CTG, MAS, TgCatBr5, TgCatBr64, TgCgCa1 and TgRsCr1. For each locus, the PCR mixture consisted of 12.5 μl PCR *Premix Taq* (TaKaRa, Dalian, China), 1 μl 10 μm forward and reverse primers, and 1.5 μl PCR-generated products in a 25 μl reaction volume. All reaction mixtures were made up to 25 μl with deionized water. The nested PCR was carried out with an annealing temperature at 60°C for 60s for all the loci. The products were digested using restriction endonucleases (Fermentas, Vilnius, Lithuania) specific for each genetic marker according to the manufacturer’s instructions. The restriction fragments were visualized by electrophoresis using a 2.5%-3% agarose gel stained with ethidium bromide and photographed using a gel documentation system (Hema, Zhuhai, China).

### Data analysis

Pearson Chi-Square and Fisher’s exact tests were used to investigate associations among qualitative categorical variables using SPSS (SPSS Inc., Chicago, Illinois). All tests were 2-sided, and the level of significant difference was defined as *P* < 0.05.

## Results

### Frequency of anti-*Toxoplasma* IgG and IgM antibodies

Data on 1014 cancer patients in age range of 27 to 91 years from six tertiary-care hospitals of Anhui province are presented in Tables 1, 2 and 3. The mean age of the participants was 57.44 ± 12.80 years old. Males constituted 61.6% and females accounted for 38.4%. Anti *T. gondii* antibodies were detectable in sera of 85 out of 1014 cases, with an overall seroprevalence of 8.38%. Among them, 61 (6.02%) patients were seropositive for only IgG, 16 (1.58%) for only IgM, and 8 (0.79%) for both IgG and IgM, with a distribution of 5.8% (6/103) in HAUCM, 11.0% (33/299) in APH, 6.6% (4/61) in HAMU, 6.0% (5/83) in XAH, 8.9% (8/90) in FHH, and 7.7% (29/378) in HBMC, respectively. No significant difference of positive rates were found in hospitals (*χ*^2^ = 4.764, *P* = 0.445), or in age groups (*χ*^2^ = 1.172, *P* = 0.947), or in gender (*χ*^2^ = 0.707, *P* = 0.400) (Tables 1 and 2).

**Table 1:** **Age-associated seroprevalence of** ***T. gondii*** **infection in cancer patients of 6 representative hospitals** ^*^

**Age group**	**HAUCM**	**APH**	**HAMU**	**XAH**	**FHH**	**HBMC**	**No. of positive/examined**	**Seroprevalence (%)**
0-39	4	28	2	8	7	23	5/72	6.9
40-49	19	73	12	27	17	82	18/230	7.8
50-59	21	68	15	12	21	94	19/231	8.2
60-69	35	78	16	30	29	103	25/291	8.6
70-79	20	43	13	3	15	65	14/159	8.8
≥80	4	9	3	3	1	11	4/31	12.9
Total	103	299	61	83	90	378	85/1014	8.38

^*^ *χ* ^2^ = 1.172, p = 0.947.

**Table 2:** **Prevalence of antibodies to** ***T. gondii*** **in cancer patients by gender in Eastern China** ^*^

**Gender**	**No. examined**	**No. positive**	**Prevalence (%)**
Male	625	56	8.96
Female	389	29	7.45
Total	1014	85	8.38

^*^ *χ* ^2^ = 0.707, p = 0.400.

**Table 3:** **The rates of positivity of** ***T. gondii*** **IgG and IgM antibodies in different cancer patients** ^*^

**Types of neoplasms**	**Number of patients**	**Positive IgG (%)**	**Positive IgM (%)**
Lung cancer	102	7 (6.86)	3 (2.94)
Gastric carcinoma	110	6 (5.45)	2 (1.82)
Rectal carcinoma	119	8 (6.72)	2 (1.68)
Intracranial tumor	13	1 (7.69)	1 (7.69)
Lymphoma	21	1 (4.76)	1 (4.76)
Breast carcinoma	56	1 (1.79)	2 (3.57)
Hepatocellular carcinoma	220	24 (10.91)	8 (3.64)
Cervical cancer	80	9 (11.25)	2 (2.50)
Carcinoma of pancreas	23	1 (4.35)	0
Esophageal carcinoma	120	6 (5.00)	2 (1.67)
Nasopharyngeal carcinoma	69	3 (4.35)	1 (1.45)
Prostatic carcinoma	13	1 (7.69)	0
Osteosarcoma	12	1 (8.33)	0

^*^ *χ* ^2^ = 16.675, p = 0.781.

The frequencies of IgG antibodies against *T. gondii* are presented in Table 3, and no statistically significant difference was seen among the cancers (*χ*^2^ = 16.675, *P* = 0.781). Additionally, the positive rate of Toxo-IgM antibodies was the highest in intracranial tumors (7.69%), followed by lymphoma (4.76%), and the lowest in nasopharyngeal carcinoma (1.45%).

### Multilocus PCR-RFLP genotyping of *T. gondii* isolates

Of the 1014 DNA samples investigated, 36 (3.55%) were positive for *T. gondii gra6* by PCR, including 3 from lung cancer, 3 from gastric carcinoma, 3 from rectal carcinoma, 1 from lymphoma, 18 from hepatocellular carcinoma, 5 from cervical cancer, 1 from oesophageal carcinoma, 1 from nasopharyngeal carcinoma, and 1 from osteosarcoma, distributed in 24 (2.37%) IgG-positive and 12 (1.18%) IgG-negative patients.

**Figure 1:**
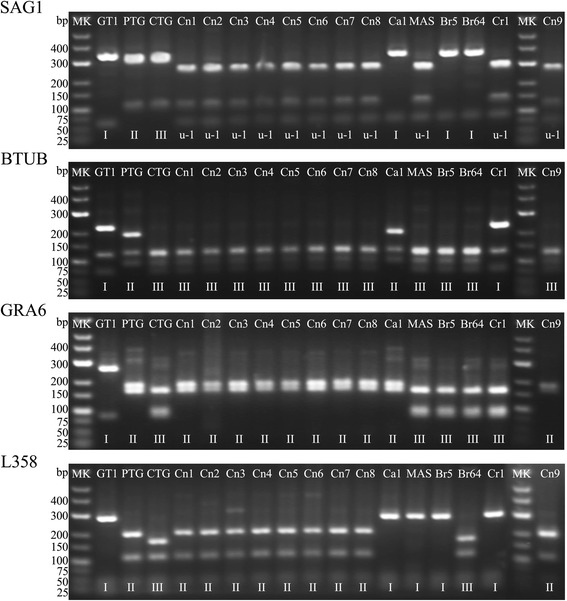
**Representative gel image of RFLP genotyping (markers SAG1, BTUB, GRA6 and L358).** Sample IDs are at the top of the gel images, genotype results are at the bottom. GT1, PTG, CTG, Ca1 (TgCgCa1), MAS, Br5 (TgCatBr5), Br64 (TgCatBr64) and Cr1 (TgRsCr1) are reference strains. Cn1: TgHuCn1; Cn2: TgHuCn2; Cn3: TgHuCn3; Cn4: TgHuCn4; Cn5: TgHuCn5; Cn6: TgHuCn6; Cn7: TgHuCn7; Cn8: TgHuCn8; Cn9: TgHuCn9. MK: molecular markers.

Nine of the 36 *gra6* positive samples showed complete genotyping results, among them 7 from hepatocellular carcinoma and 2 from cervical cancer. Only one pattern of type Chinese 1 (ToxoDB#9) was identified in the 9 samples. The electrophoresis exhibited type II pattern at *sag2*, *gra6*, *l358*, *pk1* and *c22-8*, but type III pattern at *c29-2*, *sag3*, and *btub* loci and type I pattern at the *apico* locus. The results of genotyping of *Toxoplasma* genomic DNA from humans and 8 reference strains were illustrated in Table 4 and Figure 1.

**Table 4:** ***T. gondii*** **genotypes identified in blood of PCR positive cancer patients**

**Isolate ID**	**Host**	**SAG1**	**SAG2**	**SAG3**	**BTUB**	**GRA6**	**c22-8**	**c29-2**	**L358**	**PK1**	**Apico**	**Genotype**
GT1	Goat	I	I	I	I	I	I	I	I	I	I	Reference, Type I, ToxoDB#10
PTG	Sheep	II/III	II	II	II	II	II	II	II	II	II	Reference, Type II, ToxoDB#1
CTG	Cat	II/III	III	III	III	III	III	III	III	III	III	Reference, Type III, ToxoDB#2
TgCgCa1	Cougar	Ι	II	III	II	II	II	μ-1^a^	Ι	μ-2^a^	Ι	Reference, ToxoDB#66
MAS	Human	μ-1^a^	II	III	III	III	μ-1^a^	Ι	Ι	III	Ι	Reference, ToxoDB#17
TgCatBr5	Cat	Ι	III	III	III	III	Ι	Ι	Ι	μ-1^a^	Ι	Reference, ToxoDB#19
TgCatBr64	Cat	Ι	μ-1^a^	III	III	III	μ-1^a^	Ι	III	III	Ι	Reference, ToxoDB#111
TgRsCr1	Toucan	μ-1^a^	II	III	Ι	III	μ-2^a^	Ι	Ι	III	Ι	Reference, ToxoDB#52
TgHuCn1, 2, 3, 4, 5, 6, 7, 8, 9	Human	μ-1^a^	II	III	III	II	II	III	II	II	Ι	Chinese 1, ToxoDB#9

^a^μ-1 and μ-2 represent unique RFLP genotypes, respectively.

## Discussion

The human seroprevalence of *Toxoplasma* infection is high across the world, with obvious geographical variation [26]. The majority of studies emerging from Latin American countries show significantly high rates of seropositivity, most of which had a prevalence above 60% [14]. Frequent infections are noted in Brazil from a study in pregnant women showing a prevalence of 65.1%-68.9% [27]. However, human toxoplasmosis in the United States is significantly decreased, according to the two most recent NHANES (National Health and Nutrition Examination Survey) studies, ranging from 22.5% to 12.4% [28,29]. Additionally, Rai *et al.* [30] reported a higher seroprevalence of *T. gondii* infection (68.7%) in Nepalese cancer patients than in those with ocular or other diseases. Similarly, high frequency of *T. gondii* infection has been detected among immunocompromised patients especially in those suffering from malignancy in Egypt and Korea [31,32]. In the present study, 8.38% (85/1014) of the 1014 cancer patients were seropositive for *T. gondii* tested by ELISA, which is relatively low compared with reports from other countries [33], and similar to the investigation in pregnant women of China, within the range of 0-10% [8]. The seroprevalence in humans varies in regions possibly due to the geographical factors, eating habits, pet-keeping and management, as well as differences in livestock farming practices. *T. gondii* prevalence in China was reported to be 5.2% during 1988–1992 but increased to 7.9% during 2001–2004 in two separate nationwide surveys [34], suggesting that toxoplasmosis may constitute a serious health problem in China.

The present survey did not show any significant difference in infection rates among malignancies, which differs to that reported in the previous investigation in China [6]. The high seroprevalence in cancer patients indicates a considerable risk due to the fact that the underlying latent *Toxoplasma* infection may be activated following long term chemotherapy leading to the compromised immunity of the patients. Obviously, a comparative study is needed to demonstrate the association of *Toxoplasma* infection with pathological sources of tumors and duration of chemotherapy.

In the present study, a total of 85 patients were found to be positive for antibodies against *T. gondii* (8.38%), which corresponds to 7.9% prevalence previously surveyed in the general Chinese population [35]. These results revealed that the frequency of *Toxoplasma* infection in a general hospital-based study was similar to that in a community-based study. We noted that *Toxoplasma* infection in malignant patients does not seem to increase progressively with age, which is similar to that shown with a previous study [33] but is in disagreement with the report from the United States [28]. The difference in target population surveyed may account for the varied results.

Genotyping of *Toxoplasma* isolates in humans is relatively rare due to the transient nature of parasitaemia. In toxoplasmosis, the initial dissemination of tachyzoites is usually limited to less than 20 day’s duration but this may vary according to the genotype of infected parasite and the host immune response [2]. The ability to detect *T. gondii* genomic DNA in clinical samples made it possible to directly type the infecting isolate obviating the need to harvest the parasite [36]. A nested PCR with its double amplification steps was used to improve the diagnostic yield and to allow for subsequent assessment of *T. gondii* genotypes. However, a low positive rate was found among the patients with no correlation to their serological profile. In agreement with the present results, Messaritakis *et al.* [37] tried to amplify *gra6* from clinical samples of 290 acutely infected patients and reported positive finding in only 3.12%. Since the primary objective of the present study was genotyping, GRA6 gene was selected in view of its higher polymorphism than other described markers. Understanding *T. gondii* population structure is of great interest, as it may provide us with essential information regarding the transmission and evolution of this widespread zoonotic parasite, and its pathogenesis as well. Based on 10 PCR-RFLP markers, the genetic variability of *T. gondii* isolates from China has been revealed gradually. A total of 10 genotypes were identified, indicating limited diversity of the parasite in China, which is in sharp contrast to South America where a variety of parasite lineages are transmitted in the environment [22].

It is reported that strain genotype has been associated with clinical severity of human toxoplasmosis [38]. Type II strains have been shown to be most prevalent in congenital infection and AIDS patients in North America and Europe [20,39]. In our study, only one pattern of genotype Chinese 1 (ToxoDB#9) was identified, which has been found to be widely and predominantly distributed in animal hosts such as cats, pigs, and voles in a frequency of 73.9% in China [23,40]. Our results showed that *Toxopasma* DNAs of all 9 samples share the genetic pattern of type Chinese 1, which strongly suggests the pathogenic origin of human infection from animal hosts. Type Chinese1 has also been identified from Sri Lanka, Colombia, Brazil and the United States, suggesting that it might be widespread from Asia to North and South America [24]. Obviously, more studies should be carried out for deep insight into the population genetic structure of *T. gondii* isolates and the clinical manifestations of patients and for providing useful information for control and prevention of human toxoplasmosis. To our knowledge, this is the first report of genetic typing of *T. gondii* from cancer patients in China.

## Conclusion

The 8.38% seropositive rate of *Toxoplasma* infection in Anhui province coincides with the overall prevalence in China, which is relatively low compared with that in other parts of the world. The genetic feature of *Toxoplasma* isolates found in cancer patients corroborates the findings of previous studies that *T. gondii* has a limited diversity in China. Studies on a larger number of samples from different nationalities are imperative for better understanding of the parasite genetic structure and transmission of *T. gondii* in China.

## References

[1] Dubey JP (2010). Toxoplasmosis of animals and humans.

[2] Robert-Gangneux F, Darde ML (2012). Epidemiology of and diagnostic strategies for toxoplasmosis. Clin Microbiol Rev.

[3] Hill D, Dubey JP (2002). *Toxoplasma gondii*: transmission, diagnosis and prevention. Clin Microbiol Infect.

[4] Elmore SA, Jones JL, Conrad PA, Patton S, Lindsay DS, Dubey JP (2010). *Toxoplasma gondii*: epidemiology, feline clinical aspects, and prevention. Trends Parasitol.

[5] Xu X, Liu T, Zhang A, Huo X, Luo Q, Chen Z (2012). Reactive oxygen species-triggered trophoblast apoptosis is initiated by endoplasmic reticulum stress via activation of caspase-12, CHOP, and the JNK pathway in *Toxoplasma gondii* infection in mice. Infect Immun.

[6] Yuan Z, Gao S, Liu Q, Xia X, Liu X, Liu B (2007). *Toxoplasma gondii* antibodies in cancer patients. Cancer Lett.

[7] Walle F, Kebede N, Tsegaye A, Kassa T (2013). Seroprevalence and risk factors for Toxoplasmosis in HIV infected and non-infected individuals in Bahir Dar, Northwest Ethiopia. Parasit Vectors.

[8] Gao XJ, Zhao ZJ, He ZH, Wang T, Yang TB, Chen XG (2012). *Toxoplasma gondii* infection in pregnant women in China. Parasitol.

[9] Flegr J (2013). How and why *Toxoplasma* makes us crazy. Trends Parasitol.

[10] Dubey JP (2009). History of the discovery of the life cycle of *Toxoplasma gondii*. Int J Parasitol.

[11] Allain JP, Palmer CR, Pearson G (1998). Epidemiological study of latent and recent infection by *Toxoplasma gondii* in pregnant women from a regional population in the U.K. J Infect.

[12] Dubey JP, Jones JL (2008). *Toxoplasma gondii* infection in humans and animals in the United States. Int J Parasitol.

[13] Fernandes GC, Azevedo RS, Amaku M, Yu AL, Massad E (2009). Seroepidemiology of *Toxoplasma* infection in a metropolitan region of Brazil. Epidemiol Infect.

[14] Pappas G, Roussos N, Falagas ME (2009). Toxoplasmosis snapshots: global status of *Toxoplasma gondii* seroprevalence and implications for pregnancy and congenital toxoplasmosis. Int J Parasitol.

[15] Singh S, Munawwar A, Rao S, Mehta S, Hazarika NK (2014). Serologic prevalence of *Toxoplasma gondii* in Indian women of child bearing age and effects of social and environmental factors. PLoS Negl Trop Dis.

[16] Minbaeva G, Schweiger A, Bodosheva A, Kuttubaev O, Hehl AB, Tanner I (2013). *Toxoplasma gondii* infection in Kyrgyzstan: seroprevalence, risk factor analysis, and estimate of congenital and AIDS-related toxoplasmosis. PLoS Negl Trop Dis.

[17] Vidal JE, Colombo FA, de Oliveira ACP, Focaccia R, Pereira-Chioccola VL (2004). PCR assay using cerebrospinal fluid for diagnosis of cerebral toxoplasmosis in Brazilian AIDS patients. J Clin Microbiol.

[18] Mercier A, Devillard S, Ngoubangoye B, Bonnabau H, Banuls AL, Durand P (2010). Additional haplogroups of *Toxoplasma gondii* out of Africa: population structure and mouse-virulence of strains from Gabon. PLoS Negl Trop Dis.

[19] Velmurugan GV, Dubey JP, Su C (2008). Genotyping studies of *Toxoplasma gondii* isolates from Africa revealed that the archetypal clonal lineages predominate as in North America and Europe. Vet Parasitol.

[20] Ajzenberg D, Cogne N, Paris L, Bessieres MH, Thulliez P, Filisetti D (2002). Genotype of 86 *Toxoplasma gondii* isolates associated with human congenital toxoplasmosis, and correlation with clinical findings. J Infect Dis.

[21] Khan A, Dubey JP, Su C, Ajioka JW, Rosenthal BM, Sibley LD (2011). Genetic analyses of atypical *Toxoplasma gondii* strains reveal a fourth clonal lineage in North America. Int J Parasitol.

[22] Carme B, Bissuel F, Ajzenberg D, Bouyne R, Aznar C, Demar M (2002). Severe acquired toxoplasmosis in immunocompetent adult patients in French Guiana. J Clin Microbiol.

[23] Wang L, Cheng HW, Huang KQ, Xu YH, Li YN, Du J (2013). *Toxoplasma gondii* prevalence in food animals and rodents in different regions of China: isolation, genotyping and mouse pathogenicity. Parasit Vectors.

[24] Wang L, Chen H, Liu D, Huo X, Gao J, Song X (2013). Genotypes and mouse virulence of *Toxoplasma gondii* isolates from animals and humans in China. PLoS One.

[25] Su C, Shwab EK, Zhou P, Zhu XQ, Dubey JP (2010). Moving towards an integrated approach to molecular detection and identification of *Toxoplasma gondii*. Parasitol.

[26] Flegr J, Preiss M, Klose J, Havlicek J, Vitakova M, Kodym P (2003). Decreased level of psychobiological factor novelty seeking and lower intelligence in men latently infected with the protozoan parasite *Toxoplasma gondii* Dopamine, a missing link between schizophrenia and toxoplasmosis?. Biol Psychol.

[27] Lago EG, Conrado GS, Piccoli CS, Carvalho RL, Bender AL (2009). *Toxoplasma gondii* antibody profile in HIV-infected pregnant women and the risk of congenital toxoplasmosis. Eur J Clin Microbiol.

[28] Jones JL, Kruszon-Moran D, Wilson M, McQuillan G, Navin T, McAuley JB (2001). *Toxoplasma gondii* infection in the United States: seroprevalence and risk factors. Am J Epidemiol.

[29] Jones JL, Kruszon-Moran D, Rivera H, Price C, Wilkins PP (2014). *Toxoplasma gondii* Seroprevalence in the United States 2009–2010 and Comparison with the Past Two Decades. Am J Trop Med Hyg.

[30] Rai SK, Upadhyay MP, Shrestha HG (2003). *Toxoplasma* infection in selected patients in Kathmandu, Nepal. NMCJ.

[31] Baiomy AM, Mohamed KA, Ghannam MA, Shahat SA, Al-Saadawy AS (2010). Opportunistic parasitic infections among immunocompromised Egyptian patients. J Egypt Soc Parasitol.

[32] Shin DW, Cha DY, Hua QJ, Cha GH, Lee YH (2009). Seroprevalence of *Toxoplasma gondii* infection and characteristics of seropositive patients in general hospitals in Daejeon, Korea. Korean J Parasitol.

[33] Yazar S, Yaman O, Eser B, Altuntas F, Kurnaz F, Sahin I (2004). Investigation of anti-*Toxoplasma gondii* antibodies in patients with neoplasia. J Med Microbiol.

[34] Zhou P, Chen Z, Li HL, Zheng H, He S, Lin RQ (2011). *Toxoplasma gondii* infection in humans in China. Parasit Vectors.

[35] Zhou P, Chen N, Zhang RL, Lin RQ, Zhu XQ (2008). Food-borne parasitic zoonoses in China: perspective for control. Trends Parasitol.

[36] Switaj K, Master A, Borkowski PK, Skrzypczak M, Wojciechowicz J, Zaborowski P (2006). Association of ocular toxoplasmosis with type I *Toxoplasma gondii* strains: direct genotyping from peripheral blood samples. J Clin Microbiol.

[37] Messaritakis I, Detsika M, Koliou M, Sifakis S, Antoniou M (2008). Prevalent genotypes of *Toxoplasma gondii* in pregnant women and patients from Crete and Cyprus. Am J Trop Med Hyg.

[38] Howe DK, Sibley LD (1995). *Toxoplasma gondii* comprises three clonal lineages: correlation of parasite genotype with human disease. J Infect Dis.

[39] Ajzenberg D, Yera H, Marty P, Paris L, Dalle F, Menotti J (2009). Genotype of 88 *Toxoplasma gondii* isolates associated with toxoplasmosis in immunocompromised patients and correlation with clinical findings. J Infect Dis.

[40] Li M, Mo XW, Wang L, Chen H, Luo QL, Wen HQ (2014). Phylogeny and virulence divergency analyses of *Toxoplasma gondii* isolates from China. Parasit Vectors.

